# Specifying production times in the ACT-R cognitive modeling system using evoked response potential latency

**DOI:** 10.1186/1471-2202-13-S1-P62

**Published:** 2012-07-16

**Authors:** Daniel N Cassenti, Anthony J Ries

**Affiliations:** 1 Human Research and Engineering Directorate, U.S. Army Research Laboratory, APG, MD 21005, USA

## 

**Figure 1:**
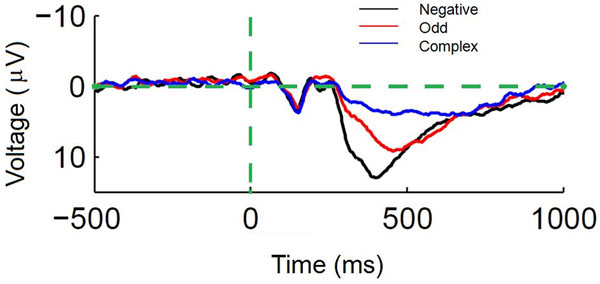
The ERP waveform at electrode site Pz, where P3 was evaluated as the latency marking 50% of the area under the P3 curve. Deciding whether a presented number was negative was least difficult and had the earliest P3 latency. Deciding whether the number was odd had middling difficulty and P3 latency. Deciding if the number was in one of two divergent parts of the number line (i.e., Complex) was most difficult and had the longest P3.

An Adaptive Control of Though Rational (ACT-R, [1]) model was built to simulate the results of an experiment that tested the relationship between brain dynamics measured with event-related potentials (ERPs) and cognitive processing associated with mathematical rule matching by having participants decide whether a presented number fit a predefined mathematical rule. Specifically, we used the latency of the N1 and P3 ERP components to assess differences in time to perceptual encoding [2] and context updating [3] respectively across three numerical rule matching conditions: Negative (press a button if the number is negative), Odd (press if the number is odd), and Complex (press if the number is negative and greater than -6 or greater than 5). First, it was hypothesized that participants would take an equal amount of time to perceptually encode a number regardless of rule difficulty and therefore N1 latency would be unchanged across levels of rule complexity. Second, it was hypothesized that P3 latency would be longer with more difficult rules since updating contextual memory cannot occur until the participant classified the stimulus as following or not following the rule. The results of the study supported these hypotheses showing that N1 latency did not change based on rule, whereas P3 latency increased with rule complexity (Figure 1).

Using the same method as had been previously established [3], model building consisted of determining the necessary productions (i.e., mental steps in ACT-R), arranging the productions, and assigning production times to simulate the timing of the cognitive correlates of the ERPs. This process specified production times in ACT-R that may be used in new models instead of relying on default and arbitrary assignment of production times.
